# Current Hospital Topics

**Published:** 1906-07-14

**Authors:** 


### HOSPITAL ADMINISTRATION.

### CONSTRUCTION AND ECONOMICS.

### CURRENT HOSPITAL TOPICS.

#### The Medical Staff Question at the Hampstead Hospital.

We are glad to note that the views we expressed on this subject in a recent issue have commended themselves to the judgment of independent medical opinion throughout the country. No one can doubt the importance of affording every practitioner of medicine the opportunity to keep in touch with modern developments by having access to hospital wards and the treatment of patients under hospital conditions. That is why the cottage hospital system has proved in practice an inestimable boon to general practitioners and their patients. As the population of suburban districts has grown, the necessity for hospitals containing more beds than the ordinary cottage hospital has arisen, and a new departure in method between the system of the larger general hospitals and the ordinary cottage hospitals is consequently called for. The Hampstead General Hospital, when completed, will contain upwards of a hundred beds, and must therefore provide facilities for the adequate treatment of every kind of case like one of the larger hospitals. For this reason it is essential that the medical staff shall not be confined to local general practitioners, but that it shall include active consultants who are allotted an adequate number of beds to enable them to do the work to the fullest requirements of the population. The King's Fund, as the representative of public and medical opinion in the highest sense, has done well to express a hope that this course will be followed by the governing authorities of the Hampstead Hospital. It is essential that the recommendations of the King's Fund shall be promptly and cordially adopted, for otherwise to attempt to proceed further with the enlargement of this hospital would be but to court financial disaster.

#### Hospital Food and Cookery.

The writer has had occasion to address many large audiences on the subject of hospitals in practically every Metropolitan division during the last few years. He has never failed to insist upon the fact that the efficiency of the modern hospital organisation is proved, from the patients' point of view, by the absence of criticism and complaints in regard to the patients' food and its preparation. The *Referee* started out to complain of hospital cooking and hospital food some weeks ago, and went so far as to state that the staple hospital diet included "half-cooked meat, vegetables floating in water, fish boiled to pulp, and flavourless milk puddings," whilst the hospitals supply "tea that is water bewitched." The statements were groundless. A hot food supply used to prove a stumbling-block to many hospitals in old days. After infinite care the difficulty has been overcome by placing the kitchen of a modern hospital in a central position and serving the food in heated trolleys with hot plates for service in the wards. Mistakes are, however, still made in rebuilding old hospitals, and St. Bartholomew's Hospital affords a striking example of this in its new out-patient block. This block is situated at one corner of the site, far away from the wards, and for some unexplained reason the new kitchen, which was badly needed, has been included in this block, so that it is highly probable that the patients' meals will not be served in the same condition of warmth and appetibility as was usual in the Military Hospital at Netley during the late war. There also the hospital kitchen was in an outhouse, and it sometimes took the attendants half an hour to wheel his dinner to an invalid. By that time it could not be very hot. Mistakes of this kind can, of course, be rectified, but they entail serious and wasteful expenditure, and do not reflect credit upon those who are responsible for them.

#### London's Street Ambulances.

It is remarkable that the *Times* should have repeatedly omitted in dealing with this question to give credit to Mr. H. L. Bischoffsheim, by whose munificence the only existing street ambulance system for London was established in 1891. Mr. Bischoffsheim provided the initial cost for supplying the ambulances, has ever since maintained the system, and paid all the outgoings attached to it, and has further stated his willingness to supply every requisite, even horse or motor ambulances, and to do anything in reason to secure to London a properly equipped, perfectly organised ambulance service. These facts were set forth in a letter to the *Times* on February 8th, 1904, and yet in every communication, including editorial articles—one published so recently as June 26th last—and in subsequent correspondence, Mr. Bischoffsheim and the existing street ambulance system are ignored entirely, although upwards of 25,000 accidents have been moved by these ambulances since their establishment. Paris, like London, owed its ambulance organisation in the first instance to private enterprise. The experience of both cities is largely identical, for, although the Paris municipality now maintains a thorough system of ambulances, it took a good number of years to merge the voluntary system in the municipality, and so to perfect and complete it. The Hospitals Association, under whose auspices the Bischoffsheim system was started, issued a manifesto and brought pressure to bear upon all candidates for the London County Council at the late election. In the result, thanks largely to Sir William Collins, assisted by Sir Shirley Murphy, the medical officer, strenuous efforts have been made this year to induce Parliament to confer power upon the London County Council to tentatively commence an ambulance system. The Bill containing these clauses has passed the House of Commons, but a Select Committee of the House of Lords, under the chairmanship of Lord Camperdown, have decided to strike out the clauses empowering the Council to establish and maintain an ambulance service from their General Powers Bill of the present session. We deal with this action elsewhere, but we may emphasise our protest of the injustice done to Mr. Bischoffsheim and his system by adding that the columns of the *Times* have contained more than one group of claimants for the honour which rightly belongs to Mr. Bischoffsheim. In fact, the *Times* claimants have mostly talked, whilst Mr. Bischoffsheim has worked and paid with a success and a liberality which entitle him to full public recognition at the hands of the press and the inhabitants of London. We all value the *Times* newspaper as a national asset, and we are confident that the failure to do justice to Mr. Bischoffsheim in its columns must be accidental, and that he will certainly receive full justice now that attention has been called to the matter.

